# Effect of face mask therapy on mandibular rotation considering initial and final vertical growth pattern: A longitudinal study

**DOI:** 10.1002/cre2.188

**Published:** 2019-06-13

**Authors:** Liseth Salazar, Melissa Piedrahita, Emery Álvarez, Adriana Santamaría, Ruben Manrique, Osmir Batista Oliveira Junior

**Affiliations:** ^1^ Department of Pediatric Dentistry, School of Dentistry CES University Medellín Colombia; ^2^ Department of Investigation and Innovation CES University Medellín Colombia; ^3^ Department of Operative Dentistry, School of dentistry University Estadual Paulista‐Unesp Araraquara Brazil

**Keywords:** Class III malocclusion, vertical growth, mandibular rotation

## Abstract

**Objectives:**

To evaluate the effect of maxillary protraction with facemask therapy on mandibular rotation taking into account the initial and final vertical growth pattern of each participant in order to evaluate our null hypothesis: The use of facemask in these patients does not modify their initial vertical growth pattern.

**Material and Methods:**

A prospective single cohort study included children with Class III malocclusion treated with rapid palatal expansion and maxillary protraction with facemask. Cephalograms were taken before commencement and after completion of the facemask therapy with standardized equipment and magnification. Intraindividual cephalometric measurements were compared, and the vertical growth patterns were classified according to cephalometric standards. Potential changes in vertical growth pattern before and after completion of the facemask therapy was assessed by measuring Pearson's chi‐square and by multiple correspondence analysis.

**Results:**

Thirty‐eight study participants were recruited, aged between 5.2 to 9.5 years (mean 7.5) at the commencement of facemask therapy, which lasted on average 1.6 years. Differences on pretherapy and posttherapy cephalograms were seen for linear rotational and sagittal measurements (*p* < .01) as well as angular measurements of the cranial base, including an average palatal plane rotation of 0.45° (standard deviation: 1.78) and an average mandibular rotation of 0.39° (standard deviation: 2.19). The majority of participants maintained their initial vertical growth pattern after facemask therapy according to the multiple correspondence analysis (*p* < .001).

**Conclusion:**

Facemask therapy does not modify vertical growth pattern. The observed changes suggest a trend of maintaining each patient's initial growth direction after therapy.

Why this paper is important

- This paper is considered the vertical growth pattern in the beginning and at the end of the therapy to be able to understand the real effect of the protraction with facial mask in the mandibular rotation.

What's known

- Hypoplasic and hyperdivergent Class III patients could be treated with this therapy because it seems to not modify their initial vertical growth pattern.

What's new

- Evaluating the effects of a therapy from different points of view enriches science and invites us to continue researching.

## 1 BACKGROUND

The skeletal Class III relationship is produced by alterations in size or position of the maxilla and jaw, presenting as retrusion or hypoplasia of the maxilla in the presence of normal jaw, as prognathism or macrognathism with normal maxilla, or as an alteration of the position and size of both jaws, involving sagittal, transversal, and vertical disharmonies (Toffol, Pavoni, Baccetti, Franchi, & Cozza, 2008; Zhang, Qu, Yu, & Zhang, 2015). Incidence of Class III malocclusion varies among populations, ranging from 1% to 5% in Whites, 5% in Latinos, and from 9% to 19% in Asians (Haynes, 1970; Thilander, 2001; Toffol et al., 2008). Additional studies report a prevalence of 3%–5% among Caucasians, 3%–6% among African‐Americans (Ngan, Hu, & Fields, 1997), and 3.7% among a Colombian population (Thilander, 2001).

This type of skeletal relationship represents one of the most challenging issues faced by the orthodontic clinician due to the unpredictable and potentially unfavorable vertical growth pattern that these patients may exhibit (Kim, Viana, Graber, Omerza, & BeGole, 1999; Zhang et al., 2015). Wolfe et al. reported that, compared with a Class I control group, patients with Class III malocclusion display hyperdivergent jaws and increased inferior facial height (Wolfe, Araujo, Behrents, & Buschang, 2011). Treatment has been aimed at maxillary advancement and/or control of jaw growth, and several devices have been designed for this purpose such as protraction face masks (FMs), chincups, mandibular cervical hedgear, and functional orthopedic appliances (Baccetti, Rey, Angel, Oberti, & McNamara, 2007; Baccetti, Rey, Oberti, Stahl, & McNamara, 2009; Baik, Jee, Lee, & Oh, 2004; Rey, Angel, Oberti, & Baccetti, 2008; Zhang et al., 2015).

Maxillary protraction with FM in conjunction with rapid palatal expansion (RPE) is a currently used therapy for Class III patients with hypoplasia or maxillary retrusion. Studies comparing treated patients with an untreated control group have shown RPE/FM therapy to be effective, especially when used at an early age (Cordasco et al., 2014; Kiliçoglu & Kirliç, 1998; Mandall et al., 2010; Nevzatoğlu & Küçükkeleş, 2014). Additionally, RPE/FM therapy has also been reported to reduce the need of subsequent surgical treatment (Mandall et al., 2016). However, counterclockwise maxillary rotation and clockwise mandibular rotation, which can be unfavorable for patients presenting clockwise vertical growth pattern, have been associated to RPE/FM treatment (Cordasco et al., 2014; Jamilian, Cannavale, Piancino, Eslami, & Perillo, 2016; Kiliçoglu & Kirliç, 1998; Nevzatoğlu & Küçükkeleş, 2014). Taking into account that RPE/FM is an effective and minimally invasive therapy for hypoplasic Class III patients, is relevant to determine whether unfavorable mandibular rotation effects are attributable to therapy, or are rather a continuation of previously established vertical growth pattern in these patients. Thus, this is a clinically relevant issue that has not been previously addressed in the literature, because studies reporting sagittal and vertical effects of FM therapy include study designs with parallel groups in that vertical growth patterns are not classified at initiation and finalization of treatment (Cordasco et al., 2014; Foersch, Jacobs, Wriedt, Hechtner, & Wehrbein, 2015).

Therefore, in this study, we assessed the effect of RPE/FM therapy on mandibular rotation taking into account the initial and final vertical growth pattern of each participant in order to evaluate our null hypothesis: the use of FM in these patients does not modify their initial vertical growth pattern.

## 2 METHODS

A prospective single cohort study was performed on Class III patients with maxillary hypoplasia treated with RPE/FM, in which a participant served as its own control. Sample size required to evaluate if the treatment would cause changes in mandibular rotation was determined to be 34 patients, using the following parameters: effect size = 0.5, statistical significance of 0.05, and power of 0.80, calculated with GpowerNT software. This study, including 38 hipoplasic Class III patients (20 females, 18 males), treated consecutively with RPE/FM in a pediatric dentistry center in Medellin, Colombia. All patients met the following inclusion criteria: anterior crossbite or edge‐to‐edge incisor relationship; no previous orthopedic treatments; prepubertal skeletal maturity CS1 and CS2 (Baccetti, Franchi, & McNamara, 2002); SNA angle smaller than 79°; Wits appraisal of 1.5 mm or less; lack of dental abnormalities (shape, size, or number), without previous extractions; and early mixed dentition with eruption of permanent incisors and first molars. Exclusion criteria were as follows: craniofacial abnormalities, psychosocial disorders that may interfere with patients' willingness to cooperate, lack of signed informed consent by parents or patient, and Class III malocclusion due to macrognathia.

In order to assess the vertical effect of FM on individual growth, we performed paired comparison analysis, considering each participant as his or her own control. Thus, each patient was classified based on his or her vertical growth pattern before and after treatment as clockwise, neutral, or counterclockwise. This was defined based on the angle formed between the mandibular plane (MP) and the sella‐nasion plane (SN), and compared with that expected for patient's age at both time points, as reported by Riolo et al. (Riolo, Moyers, McNamara, & Hunter, 1974; Table 1). Neutral vertical growth was considered in those patients whose SN/MP angle was within the expected range for patient's age, as reported by Riolo et al. Similarly, clockwise vertical growth was defined in those patients with an increased SN/MP angle, and counterclockwise vertical growth in those in which a decreased angle was identified.

**Table 1:** SN‐MP angle measurements according to age of the child, proposed as cephalometric standards by Riolo et al (1974)

Age	N	Average males	SD	Age	N	Average females	SD
7	44	36.0	4.9	7	31	36.7	4.9
8	44	35.1	4.5	8	36	35.4	5.0
9	46	34.7	4.6	9	31	35.3	5.3
10	45	34.7	4.7	10	35	35.3	5.1
11	43	34.7	4.7	11	30	34.8	5.6

Abbreviations: MP, mandibular plane; *SD*, standard deviation; SN, sella‐nasion plane.

A total of 76 conventional cephalic radiographs were taken, 38 before treatment (T1), and 38 at the end of active treatment (T2).

The Institutional Human Research Ethics Committee at CES University approved this study, Minute 109 of the Human Research Institutional Ethics Committee.

All patients had been assigned to a treatment protocol for skeletal Class III malocclusion with maxillary hypoplasia, comparable with that described by Turley (Turley, 1988), in which an RPE was performed with hyrax screw, bands in the first permanent maxillary molars, and vestibular hooks placed on canines to perform protraction with FM. When patients had a posterior crossbite, one‐quarter turn activation per day was performed for 20 days until the reached transversal relationship was appropriate for the initial condition of each patient. A protraction FM was used between 14 to 16 hr per day, with elastic bands generating 16‐ounces‐force per side (300 to 500 grams), and with an anterior and inferior force vector between 30° to 40° with respect to the occlusal plane. FM was actively used until anterior crossbite and facial aesthetic correction were achieved.

An expert operator standardized radiographic magnification factor. This same operator performed tracings and measurements on radiographs with an intraclass correlation coefficient of 0.80. Subsequently, time points T1 and T2 were superimposed for each patient by a calibrated pediatric dentist, whose results were compared with those of the expert operator, serving as the gold standard. The variables ***I/SN*** and ***PM/SN*** were used for the correlation. A high concordance between the results obtained by the expert operator and by the pediatric dentist was observed, as indicated by the intraclass correlation coefficient (***I/SN***: 0.99 and ***PM/SN***: 0.983), which showed negligible variance between lectures.

Björk's superimposition method was previously used by Wang et al. (Wang, Buschang, & Behrents, 2009), who defined “true rotation” as the angular change between the line connecting landmarks in the body of maxilla and jaw and the anterior cranial base (SN). Apparent rotation was defined as the change in the angle formed by the SN line with the mandibular and maxillary planes. The difference between true rotation and apparent rotation was defined as angular remodeling.

The following cephalometric measurements were analyzed:

N – Me: : linear measurement from nasion to menton, determines anterior facial height.
N – ANS: : linear measurement from nasion to anterior nasal spine, determines upper anterior facial height
ANS – Me: : linear measurement from anterior nasal spine to menton, determines lower anterior facial height
Gonial angle: : angular measurement formed by the mandibular plane and mandibular ramus plane
S – Go: : linear measurement from sella to gonion, determines posterior facial height
S – PNS: : linear measurement from sella to posterior nasal spine, determines posterior facial height
MP ‐S/N: : angular measurement between mandibular plane and sella‐nasion plane
IM ‐S/N– Mand: : angular measurement between the plane formed by the plane of mandibular implant simulation and sella‐nasion plane
PP ‐S/N: : angular measurement between the palatal plane and sella‐nasion plane
IM‐ S/N– Mx: : angular measurement between the plane formed by the plane of maxillary implant simulation and sella‐nasion plane
Co‐A: : linear measurement describing effective maxillary length
Co‐Gn: : linear measurement describing effective mandibular length

### 2.1 Statistical analysis

This was a before and after study design, variables were compared at T1 (before treatment) and at T2 (end of treatment). Variables were subjected to Shapiro‐Wilks normality test, which verified normality for all analyzed variables (*p* > .05). Therefore, a paired Student's t‐test was applied to compare means once the assumption of equal variances was verified. In addition, a multiple correspondence analysis (Chi^2^)(Sourial et al., 2010) was performed in order to compare the initial and final growth patterns as well as to compare the true and apparent rotations and angular remodeling.

## 3 RESULTS

A total of 38 patients (52% boys, 48% girls) treated with RPE/FM participated in this study. Average age at study initiation was 7.5 years old, with a standard deviation of 1.1 years, ranging from 5.2 to 9.5 years of age. In general, mean orthopedic treatment duration was 1.6 years, with a standard deviation of 0.5 years. At the end of the treatment, patients were of an average age of 9.1 years old, with a standard deviation of 1.1 years, ranging between 7.1 and 12 years of age.

A statistically significant increase in anterior facial height was identified when comparing linear rotational measurements at T1 and T2. Angular rotational measurements based on the anterior cranial base plane (SN), which define apparent and true rotation of both maxillae, did not exhibit significant variation (Table 2).

**Table 2:** Changes in cephalometric measurements between T1 and T2

		T1		T2		VariancesLevene's test	∆(T2‐T1)		Paired t‐testp value	Confidence interval
		$\bar{X}$	SD	$\bar{X}$	SD		$\bar{X}$	SD		
Linear rotational measurements	N ‐ Me	107.21	6.63	111.9	6.63	Equal	4.69	4.06	.00	(3.35, 6.02)
	N ‐ ANS	48.69	3.59	50.89	3.29	Equal	2.23	2.29	.00	(1.48, 2.98)
	ANS ‐ Me	60.4	4.33	62.97	4.69	Equal	2.59	2.47	.00	(1.77, 3.39)
	S ‐ Go	62.81	4.72	65.32	4.05	Equal	2.51	2.97	.00	(1.53, 3.49)
	S ‐ PNS	42.64	3.1	44.94	2.98	Equal	2.3	1.72	.00	(1.74, 2.87)
Angular rotational measurements of the cranial base	MP ‐ S/N	39.39	4.53	39.53	5.59	Equal	0.13	2.51	.75	(−0.69, 0.95)
	IM ‐ S/N ‐ Mand	39.29	4.59	39.68	5.58	Equal	0.39	2.51	.39	(−0.43, 1.21)
	PP ‐ S/N	9.26	2.91	8.82	3.18	Equal	−0.45	1.78	.13	(−1.03, 0.14)
	IM‐ S/N ‐ Mx	9.05	2.75	8.63	3.27	Equal	−0.39	2.19	.31	(−1.08, 0.35)
Linear sagittalmeasurements	Co‐A	80.14	0.71	82.08	0.63	Equal	1.94	2.68	.00	(1.06, 2.82)
	Co‐Gn	105.64	6.13	109.05	5.89	Equal	3.5	4.09	.00	(2.16, 4.85)
Other rotational measurements	Gonial angle	129.3	3.57	127.74	3.59	Equal	−1.56	1.56	.00	(−2.07, −1.04)

**Figure 1:**
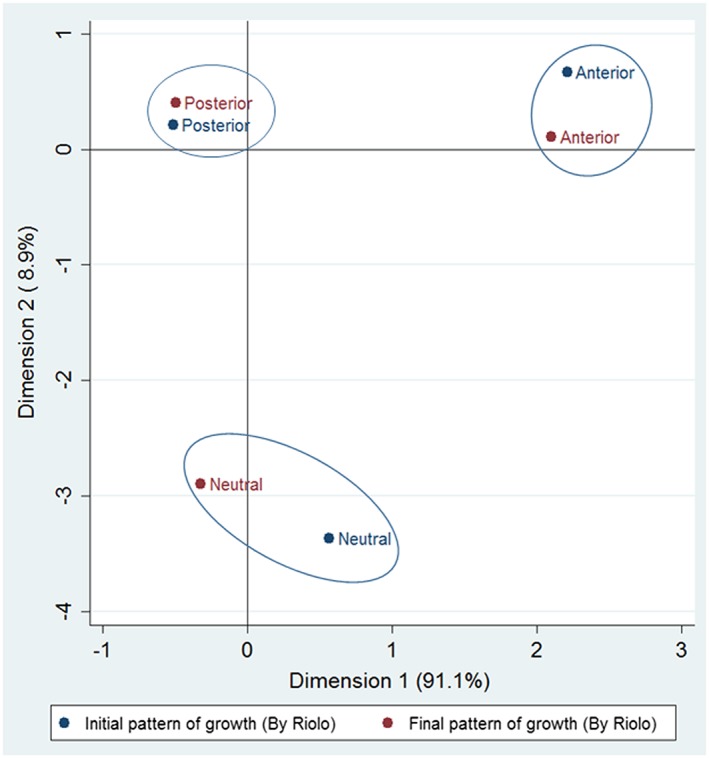
Correspondence between initial and final mandibular rotation after treatment as classified according to cephalometric standards proposed by Riolo et al (1974)

Following the guidelines from the Riolo study, correspondence analysis was performed in order to compare the initial and final vertical growth patterns for each participant. This analysis indicated that there is correspondence in all three types of growth patterns, meaning that participants maintained their vertical growth pattern after treatment regardless of whether it was clockwise, neutral, or counterclockwise (Figure 1, Table 3).

**Table 3:** Initial and final mandibular rotation after treatment (n = 38), as classified according to cephalometric standards proposed by Riolo et al (1974)

		Final mandibular rotation			Total
		Counterclockwise	Neutral	Clockwise	
Initial mandibular rotation	Counterclockwise	5	0	1	6
	Neutral	1	1	1	3
	Clockwise	1	3	25	29
Total		7	4	27	38

*Note*. Pearson's Chi2 (degrees of freedom: 4) = 23.8, *p* value = 0.000.

Correspondence analysis between true and apparent maxillary rotation showed a significant correlation between both variables (*p* < .000), thus true and apparent maxillary rotation—in the majority of cases—were in the same direction (Table 4).

**Table 4:** Correspondence between true and apparent maxillary rotation

True maxillary rotation	Apparent maxillary rotation			Total
	No rotation	Counterclockwise rotation	Clockwise rotation	
No rotation	2	2	0	4
Counterclockwise rotation	4	13	1	18
Clockwise rotation	4	0	12	16
Total	10	15	13	38

*Note*. Pearson's Chi2 (degrees of freedom: 4) = 25.8, *p* value ˂ 0.000

Apparent and true mandibular rotation measurements also exhibited a significant correlation (*p* < .001) as to the direction in that both were present, as shown in Table 5.

**Table 5:** Correspondence between true and apparent mandibular rotation

True mandibular rotation	Apparent mandibular rotation			
	No rotation	Counterclockwise rotation	Clockwise rotation	Total
No rotation	2	1	2	5
Counterclockwise rotation	1	12	1	14
Clockwise rotation	3	3	13	19
Total	6	16	16	38

*Note*. Pearson's Chi2 (degrees of freedom: 4) = 19.7, valor *p* ˂ 0.001.

## 4 DISCUSSION

In this longitudinal study, cephalic radiographs of 38 Class III malocclusion patients with maxillary hypoplasia treated with RPE/FM were analyzed. Our results suggest that by the end of treatment, the majority of patients maintain their vertical growth pattern. In agreement with previous studies (Celikoglu, Yavuz, Unal, Oktay, & Erdem, 2015; Wolfe et al., 2011), most patients presented a clockwise rotation pattern before treatment. An additional study by Guyer et al. reported that Class III patients have a more obtuse gonial angle, a longer face, and a larger MP‐S/N angle (Guyer, Ellis, McNamara, & Behrents, 1986), which is in agreement with the initial characteristics of the majority of participants included in this study.

The average palatal plane rotation was −0.45 degrees (*p* = .13), and the average MP rotation was 0.39 degrees (*p* = .39), which were not considered statistically significant. Direction of maxillomandibular rotation was confirmed by using the superimposition method described by Bjork, and in addition, the direction of the true and apparent rotations coincided on both jaws. In contrast, previous studies reported statistically significant changes in the maxillary and MPs, indicative of counterclockwise maxillary rotation and clockwise mandibular rotation after treatment (Cordasco et al., 2014; Foersch et al., 2015; Liu, Zhou, Wang, Liu, & Zhou, 2015). Those studies included designs in that two parallel groups are analyzed, and behavior of each individual' vertical growth pattern was not evaluated (Cordasco et al., 2014; Foersch et al., 2015; Liu et al., 2015; Seehra, Fleming, Mandall, & Dibiase, 2012). In our study, a paired design with an appropriate sample size was considered as the best option to establish whether the mandibular rotational effects attributed to this therapy are a consequence of an alteration in initial vertical growth pattern, or a continuance of the growth trend established on these patients.

By the end of the treatment, the majority of patients maintained their initial vertical growth pattern, showing some changes in growth direction, mainly in those with neutral rotation. Of the former, some patients shifted toward having clockwise growth direction, whereas others had counterclockwise growth direction; suggesting that in response to therapy, changes toward clockwise mandibular rotations should not always be expected, as previously reported by others (Kiliçoglu & Kirliç, 1998; Nevzatoğlu & Küçükkeleş, 2014; Wolfe et al., 2011).

Data were analyzed from three different perspectives: assessment of changes in T1 and T2, confirmation of results by Bjork superimposition method, and finally, taking into account each patient's vertical growth pattern. By using these three approaches, we were able to confirm that although there are changes in mandibular rotation after therapy, these appear to be a continuation of the patient's growth pattern rather than a side effect of FM therapy. Yifan Lin et al. evaluated to stability of maxillary protraction therapy in children with Class III malocclusion in a systematic review and meta‐analysis (Lin, Guo, Hou, Fu, & Li, 2018) and report that in the short‐term treatment, the angle of the MP increases 1.41°, but in the posttreatment changes, it presents a decrease of −0.89°. These results show that the effect of posterior mandibular rotation attributed to therapy must be assumed with caution as our results show. Understanding that vertical effects associated to FM therapy are more related to individual growth pattern rather than to therapy itself is a clinically relevant issue because it opens the possibility of using this therapy in Class III malocclusion patients with initial clockwise vertical growth pattern that require maxillary advancement due to their facial characteristics.

Protraction FM therapy has been shown to be the best therapeutic option for treatment of Class III malocclusion at an early age (Woon & Thiruvenkatachari, 2017), but its use has been limited in patients with clockwise vertical facial patterns, thus reducing their number of therapeutic options even if there is a need for maxillary advancement.

Altogether, our results strongly suggest that FM therapy could be used in Class III malocclusion patients with clockwise vertical growth, whom were up to date not considered candidates for this type of therapy. Use of FM/RPE therapy in these patients may improve prognosis as well as reduce the need of future aesthetic and functional surgical corrections. Furthermore, we propose that studies on maxillary protraction with FM therapy on growing patients should always include the initial assessment of each patient's rotation pattern in order to precisely evaluate the effect of treatment on growth, and thus confirm the potential clinical impact of our observations.

## 5 CONCLUSIONS

Our results suggest that FM therapy does not modify vertical growth pattern. The changes in mandibular rotation observed after therapy tend to maintain each patient's initial vertical growth pattern because the majority of patients conserved the same vertical growth pattern at the end of therapy.

## AUTHOR CONTRIBUTIONS

All the members of the team were in charge of the design of the study. EA and AS collected the sample. LS and MP performed the tracing and cephalometric analysis. RM and OB performed the statistical analysis, and every member of the team participated in the analysis of the results and in the discussion. All authors read and approved the final manuscript.
